# Prediction of response to therapy with ezatiostat in lower risk myelodysplastic syndrome

**DOI:** 10.1186/1756-8722-5-20

**Published:** 2012-05-06

**Authors:** Naomi Galili, Pablo Tamayo, Olga B Botvinnik, Jill P Mesirov, Margarita R Brooks, Gail Brown, Azra Raza

**Affiliations:** 1 Department of Medicine, Division of Hematology and Oncology, Columbia University Medical Center and New York Presbyterian Hospital, 177 Fort Washington Ave., New York, NY, 10032, USA; 2 The Eli and Edythe L. Broad Institute of Massachusetts Institute of Technology and Harvard University, 301 Binney Street, Cambridge, Massachusetts, 02142, USA; 3 Telik, Inc, 300 Hanson Way, Palo Alto, CA, 94304, USA

## Abstract

**Background:**

Approximately 70% of all patients with myelodysplastic syndrome (MDS) present with lower-risk disease. Some of these patients will initially respond to treatment with growth factors to improve anemia but will eventually cease to respond, while others will be resistant to growth factor therapy. Eventually, all lower-risk MDS patients require multiple transfusions and long-term therapy. While some patients may respond briefly to hypomethylating agents or lenalidomide, the majority will not, and new therapeutic options are needed for these lower-risk patients. Our previous clinical trials with ezatiostat (ezatiostat hydrochloride, Telentra®, TLK199), a glutathione S-transferase P1-1 inhibitor in clinical development for the treatment of low- to intermediate-risk MDS, have shown significant clinical activity, including multilineage responses as well as durable red-blood-cell transfusion independence. It would be of significant clinical benefit to be able to identify patients most likely to respond to ezatiostat before therapy is initiated. We have previously shown that by using gene expression profiling and grouping by response, it is possible to construct a predictive score that indicates the likelihood that patients without deletion 5q will respond to lenalidomide. The success of that study was based in part on the fact that the profile for response was linked to the biology of the disease.

**Methods:**

RNA was available on 30 patients enrolled in the trial and analyzed for gene expression on the Illumina HT12v4 whole genome array according to the manufacturer’s protocol. Gene marker analysis was performed. The selection of genes associated with the responders (R) vs. non-responders (NR) phenotype was obtained using a normalized and rescaled mutual information score (NMI).

**Conclusions:**

We have shown that an ezatiostat response profile contains two miRNAs that regulate expression of genes known to be implicated in MDS disease pathology. Remarkably, pathway analysis of the response profile revealed that the genes comprising the jun-N-terminal kinase/c-Jun molecular pathway, which is known to be activated by ezatiostat, are under-expressed in patients who respond and over-expressed in patients who were non-responders to the drug, suggesting that both the biology of the disease and the molecular mechanism of action of the drug are positively correlated.

## Background

Myelodysplastic syndrome (MDS) is a clonal stem cell disorder resulting in bone marrow failure and variable cytopenias. Development of new treatment strategies has greatly improved the outlook for patients with MDS. There are three FDA-approved drugs for therapy of patients who have become transfusion-dependent, including two hypomethylating drugs (HMAs), azacitidine and decitabine, and the thalidomide derivative lenalidomide. Patients with higher-risk disease have been shown to benefit from HMA therapy [1,2], while patients with lower-risk disease with a karyotype of clonally restricted deletion of the long arm of chromosome 5 (deletion 5q or del[5q]) are highly responsive to lenalidomide [3,4]. Only 26% of transfusion-dependent lower-risk patients without del(5q) will also become transfusion-independent while on treatment [5], but the FDA has not approved lenalidomide for these patients. There are few treatment options for the majority of transfusion-dependent MDS patients with lower-risk disease. This situation represents a significant unmet medical need. Once disease-modifying therapy is required by the patient, it is a challenge for the treating physician to decide which drug will best benefit the individual patient, as only a subset responds to any given agent.

Ezatiostat (ezatiostat hydrochloride, Telintra®, TLK199), a glutathione analog inhibitor of the enzyme glutathione *S*-transferase P1-1 (GSTP1-1), causes dissociation of the enzyme from the jun-N-terminal kinase/c-Jun (JNK/JUN) complex, leading to JNK activation by phosphorylation. Activated JNK phosphorylates c-JUN, which ultimately results in the stimulation of all myeloid lineages hematopoietic progenitor’s proliferation and maturation. In addition, subsequent activation of the caspase-dependent apoptotic pathway increases reactive oxygen species in human leukemia blast cells. This cascade can trigger apoptosis. In other words, the therapeutic action of ezatiostat appears to include both proliferation of normal myeloid progenitors as well as apoptosis of the malignant clone.

Our previous phase 2 study of ezatiostat demonstrated that this drug can elicit a therapeutic response in a proportion of patients with lower-risk MDS [6]. Trilineage responses were observed in 4 of 16 patients (25%) with trilineage cytopenia. Hematologic Improvement-Erythroid (HI-E) was observed in 9 of 38 patients (24%), HI-Neutrophil (HI-N) in 11 of 26 patients (42%), and HI-Platelet (HI-P) in 12 of 24 patients (50%). In a subgroup of 9 patients who were red-blood-cell (RBC)-transfusion-dependent and HMA-naïve, a 47% HI-E rate was observed. Three (16%) of these patients achieved complete RBC-transfusion independence, and 3 of 9 (33%) reported multilineage responses. While the responses seen in the lower-risk patients resulted in hematologic improvement with clinically significant reductions in RBC-transfusion requirements, and in some cases transfusion-independence, it is clear that in this heterogeneous disease it would be advantageous if a diagnostic predictor of response could be developed to optimize treatment outcomes.

Gene expression profiling studies can define signatures that are capable of improving existing classification and prognosis of multiple diseases, especially malignancies which tend to be heterogeneous or of unknown or uncertain origin. MDS is a group of hematopoietic stem cell disorders that pose a unique challenge for gene expression profiling by virtue of their inherent heterogeneity. However, we have previously shown that profiling can generate distinct expression signatures based on the uniform grouping of patient response to a specific drug therapy [7]. In an attempt to identify the subset of lower-risk patients likely to benefit from therapy with ezatiostat, we examined pre-therapy marrow cells from ezatiostat-treated MDS patients by gene expression profiling in order to identify signatures which differentiate responders from non-responders.

## Methods

### Patient samples

A separate research protocol was submitted to the institutional review boards (IRBs) at the University of Massachusetts Memorial Medical Center, Worcester, MA, and at Saint Vincent’s Comprehensive Cancer Center, New York, NY, seeking permission to perform the microarray analysis as described below. Once the research protocol was approved by the respective IRBs and informed consent was obtained from each patient, samples from lower-risk MDS patients treated with ezatiostat in the phase 2 clinical trial at those institutions were obtained. Mononuclear cells from pre-therapy bone marrow aspirates were stored in Trizol at °C.

All patients had low- or intermediate-1-risk MDS as determined by the International Prognostic Scoring System (IPSS) and had not received growth factors for 4 weeks prior to study enrollment. Hematological improvement response was based on International Working Group (IWG) 2006 criteria [8,9].

### Microarrays

Total RNA was purified from 5-10 × 10^6^ mononuclear cells using Trizol (Invitrogen) and analyzed for gene expression on the Illumina HT12v4 whole genome array according to the manufacturer’s protocol. RNA was available on 30 patients enrolled in the trial at the two institutions.

### Gene marker analysis

The selection of genes associated with the responders (R) vs. non-responders (NR) phenotype was obtained using a normalized and rescaled mutual information (NMI) score. This quantity was obtained using a kernel-density-based estimate of the joint probability and the mutual information distribution between the phenotype and each gene profile. The resulting mutual information [10] was then normalized by the joint-entropy in order to provide a more universal metric [11], rescaled to the interval (0, 1), and assigned a “directionality” sign defined according to the sign of the Pearson correlation between the phenotype and the gene profile. A perfect gene-phenotype match (anti-match) using this NMI score corresponds to a +1(−1) value, and a random match attains approximately 0. This quantity as a metric for gene selection has advantages over the Pearson correlation and other more traditional two-sample tests, such as its increased sensitivity and wider high-score dynamic range to detect nonlinear relationships, and its capability to match continuous or/and discrete profiles and phenotypes. The significance of a given NMI score is typically estimated by a permutation test where the values of the phenotype are randomly permutated many times, and a nominal *p*-value is computed according to how many times the matching scores of the random permutations are more extreme than the actual score. Because in our study the number of samples is small, we opted for not performing the permutation test and focused instead on analyzing the 100 genes with the highest (50) and lowest (50) NMI scores.

### Gene set/pathway analysis

To project the gene profiles into the space of pathways, we used a single-sample Gene Set Enrichment Analysis (ssGSEA) [12-17]. The gene-expression values were first rank-normalized and sorted independently, sample per sample. Then a per-gene enrichment score for each gene set/pathway was computed based on the integrated difference between the empirical cumulative distribution functions of: i) the genes in the gene set vs. ii) the genes not in the set. This procedure is similar to the computation of standard Gene Set Enrichment Analysis [13], but it is based on absolute rather than differential expression.

The selection of gene sets/pathways more associated with the responders vs. non-responders phenotype was obtained using an NMI as was done with the gene profiles (see above paragraph). The sources of gene sets/pathways were: i) the C2 sub-collection of curated and functional gene sets from the Molecular Signatures Database (MSigDB) release 2.5 (http://www.broadinstitute.org/msigdb); ii) an internal database of signatures of oncogene activation containing over 300 gene sets defined from data generated in our laboratory, from GEO datasets, and from the biomedical literature; and iii) gene sets representing hematopoietic cell populations [18]. We considered a total of 2776 gene sets. The selection analysis was restricted to the 60 gene sets/pathways with the 30 highest and 30 lowest NMI scores.

## Results and discussion

In order to identify an expression signature of ezatiostat response, prior to therapy with the drug, the genome-wide gene expression profiles of bone marrow aspirate mononuclear cells were obtained from patients with MDS. Samples of nine responders and 21 non-responders were available for analysis. The nine responders included one with a baseline single erythroid cytopenia, one with a single platelet cytopenia, one with erythroid-neutrophil cytopenias, two with erythroid-platelet cytopenias, two with neutrophil-platelet cytopenias and two with trilineage cytopenia (Table 1). The non-responders included 11 patients with a single erythroid cytopenia, one with single platelet cytopenia, one with single netrophil cytopenia, two with erythroid-platelet cytopenias, two with erythroid-neutrophil cytopenias, and one with trilineage cytopenias. There were 18 patients with refractory anemia (RA); eight with RA with ringed sideroblasts (RARS); three with RA with excess blasts, type 1 (RAEB-1); and one with RAEB-2. Patient samples had similar representation in both the responder and the non-responder groups (Table 1).

**Table 1:** Myelodysplastic syndrome disease characteristics of patients treated with ezatiostat and analyzed by Illumina expression arrays

**Baseline Cytopenia**	**Illumina #**	**Response**	**Sex**	**Age**	**Hgb**	**WBC**	**ANC**	**Platelets**	**WHO**	**IPSS**	**Blasts (%)**	**Karyotype**
E only	TLK-1	NR	F	69	9.3	4.4	3.08	325	RARS	Int-1	1	46,XX,del(11)(q14q23)[11]/46,XX[9]
E only	TLK-10	NR	F	84	7.7	3.7	2.07	324	RAEB-2	Int-1	10	NA
E only	TLK-11	NR	M	53	11	9.7	7,00	310	RA	Int-1	2	47,XY[19]
E only	TLK-12	NR	M	81	8.9	4..1	2.10	343	RA	Low	1	45,X,-Y[4]/46,XY[16]
EN	TLK-13	NR	F	72	10	1.6	0.58	113	RAEB-1	Int-1	6	46,XX[19]
P only	TLK-14	NR	M	76	12	1.7	1.27	94	RA	Low	1	45,X,-Y[19]
E only	TLK-15	NR	F	67	6.2	1.8	1.08	133	RA	Low	3-5	46,XX[19]
NP	TLK-16	NR	F	68	12.2	3.2	1.05	73	RA	Low	<5	46,XX[19]
EN	TLK-2	NR	M	67	10	2.8	1.232	120	RA	Int-1	1	46,XY[20]
EP	TLK-21	NR	M	63	10	3.2	0.64	76	RA	Int-1	<5	46,XY[19]
E only	TLK-27	NR	F	73	9.2	4.6	3.22	221	RARS	Int-1	0-1	46,XX,der(7)t(1;7)(q25;36) ort(1;7)(q23;q32)[5]/46,Xdel(X)(q24q28),der(7)t(1;7)(q25;q36) ort(1;7)(q23;q32)[15]
N only	TLK-28	NR	M	67	14	3.2	0.99	108	RARS	NA	NA	46,XY[19]
ENP	TLK-29	NR	F	74	10.8	3.7	1.27	56	RA	Int-1	1	46,XX,dup(1)(q21q42)[16]/46,XX[4]
E only	TLK-3	NR	M	71	8.9	2.1	1.39	108	RA	Low	0-1	46,XY[19]
EP	TLK-30	NR	M	79	10	3.17	3.36	101	RA	Low	1	46,XY,del(20)(q11.2q13.3)[19]
P only	TLK-4	NR	M	77	13	3.3	2.25	93	RA	Int-1	0	46,XY-20,+/der(20)del(20)(p12)del(20) (q11.2)[21]
E only	TLK-5	NR	M	81	7.8	10.8	7.88	157	RARS	Low	2	46,XY,del(2)(p13),inv(5)(q13q33),add(13)(q22)[7]/46,XY,del(5)(q13q33)[3],/add(13)(q22)[3]/46,XY[10]
E only	TLK-6	NR	M	73	10	3.9	3	289	RA	Low	1	46,XY[20]
E only	TLK-7	NR	F	67	11	8.3	3.74	251	RARS	Int-1	1-2	47,XX,+8[14]/46,XX[6]
NP	TLK-8	NR	M	75	12	2.9	1.22	104	RA	Int-1	1	44,XY,del(3)(p12p21),-5-9,add(13)(p11.2)[5]/44,X,-Y,del(3)(p12p21),del(5)(q13q33),add (6)(q21),-9,-12,add(13)(p11.2),+mar[2]/43,sdl118 [2]/43,sdl2,add(7)(q22)[6]/46,XY[5]
E only	TLK-9	NR	M	77	9.1	4	2.6	253	RARS	Low	1	46,XY[19]
ENP	TLK-17	R	F	66	8.7	2	0.34	90	RARS	Int1	3	46,XX[19]
E only	TLK-18	R	M	80	9.3	2.7	1.9	168	RA	Low	4	46,XY[19]
ENP	TLK-19	R	M	79	10	3.3..	0.79	98	RAEB-1	Int-1	4	46,XY,t(2;3)(p15;q27)[11]/46,XY [9]
EN	TLK-20	R	M	68	9.7	2.8	0.67	336	RARS	Low	2	46,XY[19]
P only	TLK-22	R	M	71	11	5.64	3.38	35	RA	Low	1	46,XY[19]
NP	TLK-23	R	F	71	13	2.6	1.48	106	RA	Int-1	0-1	46,XX,-9,del(11)(q13),+mar[22]/46,XX[2]
EP	TLK-24	R	M	81	9.2	5.6	3.02	148	RA	Low	1	45,X,-Y[11]/46,XY[9]
EP	TLK-25	R	M	79	11	3.7	1	68	RAEB-1	Int-1	5	46,XY[19]
NP	TLK-26	R	F	75	13	3.6	1.26	39	RA	Low	3	46,XX[19]

Abbreviations: *ANC* absolute neutrophil count, *E* erythroid, *F* female, *Hgb* hemoglobin, *Int-1* International-1, *IPSS* International Prognostic Scoring System, *M* male, *N* neutrophil, *NR* nonresponder, *P* platelet, *R* responder, *RA* refractory anemia, *RAEB-1* refractory anemia with excess blasts type 1, *RAEB-2* RA with excess blasts type 2, *RARS* refractory anemia with ringed sideroblasts, *WBC* white blood count, *WHO* World Health Organization.

**Figure 1:**
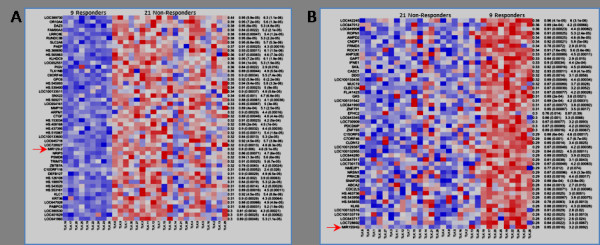
Patients who responded to ezatiostat under-expressed miR-129 (A) and over-expressed miR-155 (B).

We compared the gene expression profiles of responders and non-responders to identify genes that correlate with ezatiostat response. The top 100 marker genes (50 under-expressed and 50 over-expressed in the responders) were identified using a sensitive metric based on the normal mutual information (NMI; see Methods) (Figure 1A and B). A majority of the top genes in both profiles are transcripts of as-yet unknown function. Most notably, however, there are two microRNA (miR) genes that are differentially expressed. Responders under-express miR-129 and over-express miR-155. miRNAs are small non-coding RNAs of 18–25 nucleotides that bind the 3’ UTR of mRNA, resulting in suppressed translation or mRNA degradation. This post-transcriptional control has been found to be perturbed in a wide variety of tumors, where it has been shown to have both oncogenic and tumor-suppressor activities [19]. Surprisingly, both miRNAs have been shown to mediate control of molecular pathways associated with the pathophysiology of MDS.

Reduced expression of miR-129 has been found in a variety of primary solid tumors and has been shown to reduce proliferation by targeting the G → S cell cycle kinase CDK6 in lung epithelial-derived cells [20]. Interestingly, one of the direct targets of miR-129 is the oncogene SOX4, a member of the SRY-related high mobility group box family of transcription factors [21]. Over-expression of SOX4 has been demonstrated in prostate, liver, lung, bladder, and medulloblastoma cancers exhibiting poor prognosis [21]. SOX4 has also been shown to target growth factor receptors that when stimulated increase proliferation as well as inhibit differentiation via suppression of other transcription factors [22]. Supporting the role of SOX4 in myeloid cells are the in vitro studies showing that over-expression of SOX4 in 32D cells resulted in the suppression of cytokine-induced granulocyte differentiation [23]. Other predicted target genes are components of the RISC complex that processes miRNAs from their precursor molecules [22]. Thus the low expression of miR129 seen in responders would be expected to aberrantly affect proliferation and differentiation and, through dysregulated miRNA processing, to participate in oncogenic transformation. These are precisely the pathways that are associated with the evolution of MDS.

In contrast, miR-155 is over-expressed in patients who responded to ezatiostat. miR-155 has been previously shown to be over-expressed in bone marrow cells of patients with acute myelogenous leukemia (AML) [24]. Recently, c-MYB was shown to bind to the promoter region of the gene for miR-155 and to stimulate its transcription in B-cell chronic lymphocytic leukemia [25]. It may be that in MDS as well, MYB stimulates expression of miR155. This observation is especially important in light of recent studies that showed that forced expression of miR-155 in mouse hematopoietic marrow cells results in granulocyte/monocyte expansion, with these cells having dysplastic features [24]. This proliferation was accompanied by decreased erythrocytes, megakaryocytes, and lymphocytes in the marrow. In addition, when expression analysis was performed on the marrow cells, genes known to be important for normal hematopoiesis were found to be down-regulated.

**Figure 2:**
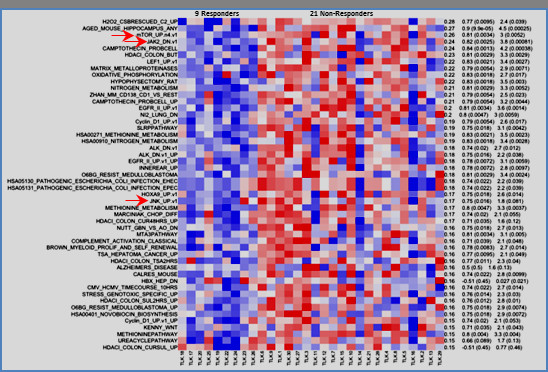
Three pathways, mTOR, JAK2, and JNK, were under-expressed in patients who responded to ezatiostat.

Single-sample Gene Set Enrichment Analysis was performed to find the most salient differences in terms of pathways and biological processes between responders and non-responders. Most notably, three pathways, mTOR, JAK2 and JNK, were all found to be under-expressed in the responders (Figure 2). All three have significant implications in the process of hematopoiesis.

The serine/threonine kinase Akt is the upstream regulator of mTOR and functions as an antiapoptotic kinase. AKT is the major downstream target of PI3K (phophoinositide-3 kinase), which may be activated by receptor tyrosine kinases (RTKs), including epidermal growth factor receptor (EGFR), insulin-like growth factor-1 receptor (IGF-1R), and G protein-coupled receptors (GPCRs). It has been shown that the PI3K/Akt/mTOR pathway is activated in high-risk MDS, when compared to lower risk or healthy controls [26]. In addition, mTOR was specifically shown to be upregulated in the myeloid progenitors of high-risk MDS. These results suggest that this pathway participates in the evolution of MDS and that patients with low expression of these genes may respond to ezatiostat. JAK2 (tyrosine Janus Kinase-2) is an important regulator of erythropoiesis. When erythropoietin binds to its receptor on progenitor cells, the receptor forms homodimers that physically associate with JAK2, resulting in phosphorylation and activation. The activated tyrosine residues then associate with multiple downstream adaptors and effectors, including PI3K and JNK [27,28]. The resulting effects are promotion of erythroid differentiation and the synthesis of hemoglobin. As with the mTOR pathway, those patients able to respond to ezatiostat appear to be those who under-express genes of the JAK2 activation pathway.

**Figure 3:**
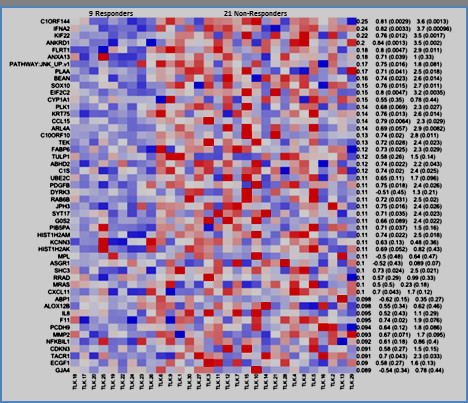
The gene-set profile of JNK-inhibited keratinocytes is similar to the gene-set profile of patients who respond to ezatiostat.

Lastly, and most striking, was the finding that the JNK/JUN pathway, which has been shown to be central to ezatiostat’s molecular mechanism of action, is also under-expressed in responding patients. This gene set, as defined by the GEO dataset GDS2081, was derived from expression studies in primary cultured human epidermal keratinocytes, with activated JNK/JUN exposed to the JNK inhibitor drug SP600125 and analyzed on Affymetrix HGU95Av2 arrays [29]. A heatmap of responders/non-responders was derived from the combined enrichment score of the top/bottom 200 genes, of which the top expressing genes are shown in Figure 3. Most notably, the gene-set profile of the JNK-inhibited keratinocytes is highly similar to the gene-set profile of patients who respond to ezatiostat. In other words, the profile is the same when the JNK pathway is dysregulated in vitro by the drug or pathologically, as in some MDS patients. Ezatiostat has been shown to activate the JNK/JUN pathway; thus it is reasonable to expect that patients whose pre-treatment marrow cells show low expression will respond to ezatiostat therapy. In contrast, we show here that patients whose cells do not under-express the JNK/JUN pathway are not likely to benefit from additional activation by ezatiostat.

In conclusion, a bedside-to-bench strategy correlating MDS patient pre-treatment genomic data with clinical response to ezatiostat has yielded positive markers for this investigational drug’s clinical efficacy. These signature genes and signaling pathways positively correlate with the known mechanism of action of ezatiostat. The genomic signature reported herein that distinguishes responders from non-responders among MDS patients treated with ezatiostat may enable the future selection of patients who are most likely to positively benefit from ezatiostat treatment. These markers could potentially be developed into a clinical diagnostic test for MDS patient sensitivity to ezatiostat treatment.

## Competing interests

Gail Brown is an employee of Telik, Inc. Azra Raza has received honoraria from Celgene Corporation to serve on their speakers’ bureau. Naomi Galili, Pablo Tamayo, Olga B Botvinnik, Jill P Mesirov and Margarita Roserika Brooks declare that they have no competing interests.

## Authors’ contributions

NG conceived the study, obtained the Illumina results, and wrote the manuscript. PT, OBB and JPM did the computational analysis and generated the figures. MRB coolected the clinical characteristics and made Table 1. GB and AR conceived the study, assigned the clinical response criteria and participated in the writing of the manuscript. All authors read and approved the final manuscript.
